# Unequal Access, Unequal Outcomes: Analyzing Chronic Obstructive Pulmonary Disease Hospital Admissions Across Clinical Commissioning Groups

**DOI:** 10.1016/j.opresp.2025.100524

**Published:** 2025-11-21

**Authors:** Sushant Sharma

**Affiliations:** Faculty of Medical and Health Sciences, University of Auckland, 85 Park Road, Grafton, Auckland 1023, New Zealand

**Keywords:** Chronic obstructive pulmonary disease, Hospital admissions, Geographic variation, Health inequalities, Primary care access, England, Enfermedad pulmonar obstructiva crónica, Hospitalizaciones, Variación geográfica, Desigualdad en materia de salud, Acceso a la atención primaria, Inglaterra

## Abstract

Chronic obstructive pulmonary disease (COPD) remains a major cause of hospitalisation and mortality in England, with outcomes differing widely across Clinical Commissioning Groups (CCGs). This short communication highlights regional variation in COPD emergency admissions and 30 day readmission patterns using figures from the 2nd Atlas of Variation in Risk Factors and Healthcare for Respiratory Disease by Public Health England. Northern regions show higher rates than southern regions, reflecting variation in access to primary care, respiratory specialists, and pulmonary rehabilitation. These patterns indicate that supply sensitive factors contribute significantly to unequal outcomes. Reducing avoidable hospitalisations will require fairer resource allocation, earlier community based management, and stronger coordination between hospital and primary care services.

## Abstract (es)

En Inglaterra, la enfermedad pulmonar obstructiva crónica (EPOC) sigue siendo un problema relevante de salud pública, y representa gran parte de las hospitalizaciones y de las tasas de mortalidad. Debido sobre todo a las diferencias en el acceso a la atención sanitaria, en la asignación de recursos y en la aplicación de estrategias preventivas, los resultados relativos a la EPOC tienen una marcada heterogeneidad geográfica. Este artículo breve destaca las diferencias existentes y examina cómo se ve afectado el manejo de la EPOC por las disparidades en la atención sanitaria sensible a la oferta, entre distintas áreas sanitarias de gestión. Se recalca la necesidad de hacer un reparto asistencial justo para reducir la dependencia respecto de los hospitales y mejorar los resultados a largo plazo, evaluando elementos importantes como la accesibilidad a la atención primaria, la disponibilidad de especialistas y la formulación de políticas.

## Introduction

In England, chronic obstructive pulmonary disease (COPD) continues to be a significant public health concern, accounting for a large portion of hospital admissions and death rates. Due mainly to variations in healthcare access, resource allocation, and the application of preventative strategies, there is significant geographical heterogeneity in COPD-related outcomes.^1^ This issue brief underlines the existing variations and examines how supply-sensitive care variation affects COPD management across Clinical Commissioning Groups (CCGs). This brief emphasizes the necessity of fair healthcare distribution to lower hospital reliance and enhance long-term results by assessing important elements such as primary care accessibility, specialist availability, and policy actions.^2^

In England, COPD is a significant source of morbidity and mortality, and hospital admissions and healthcare usage vary significantly by location.^3^ These disparities are not merely reflective of differences in disease prevalence but also indicate variations in healthcare access and delivery. Regardless of clinical need, the availability of healthcare resources affects patient outcomes, according to the theory of supply-sensitive care variation.^4^

This problem is significant when managing COPD, as areas with better access to specialists and more integrated primary care typically have lower hospitalization rates and better health outcomes.^5^ In order to create focused policy measures that guarantee fair healthcare delivery, these variations must be addressed.

## Geographic inequalities in COPD admissions and healthcare access

The pronounced regional variations in COPD hospitalization rates demonstrate the influence of healthcare resource allocation. According to data, the South West has a much lower emergency admission rate of 112.1 per 100,000, whereas the North West has a rate of 624.96 per 100,000.^6^ The discrepancy is also evident in 30-day readmission rates, which show greater readmission frequency in areas with less follow-up care.^7^ These variations imply that systemic problems in healthcare delivery and illness load have a role in these outcomes.

## The 30-day readmission rates for COPD patients in England's Clinical

Commissioning Groups (CCGs) (2017/18) are shown in Table 1 to help clarify this issue. Significant disparity can be seen in the statistics, with the North West having the highest readmission rate (22.3%) and the South West having the lowest rate (5.9%).^1^, ^6^ These differences imply that areas with greater readmission rates might not have access to enough post-hospitalization treatment, such as pulmonary rehabilitation programs, follow-up primary care visits, and expert consultations.^6^ Furthermore, high readmission rates frequently suggest a higher dependence on hospital-based treatment, suggesting possible flaws in long-term disease control plans and outpatient management. Better coordination between hospital and primary care clinicians, more robust community-based treatments, and improved discharge planning are all necessary to address these disparities.^7^

**Table 1:** COPD 30-day hospital readmission rates across Clinical Commissioning Groups (CCGs) in England, 2017/18.

CCG region	30-Day readmission rate (%)
North West	22.3
North East	19.8
Yorkshire & Humber	18.4
West Midlands	16.5
East Midlands	14.7
East of England	12.9
London	10.5
South East	9.2
South West	5.9

One key factor influencing the course of COPD is access to primary care. Due to delayed diagnosis and inadequate illness care, emergency hospital visits are frequently more significant in areas with fewer general practitioners and respiratory specialists.^8^ Disparities are exacerbated by the unequal implementation of preventive care methods across areas, such as early pulmonary rehabilitation and smoking cessation programs.^9^ With some CCGs enforcing higher referral requirements that unintentionally increase hospital dependence, delayed specialist referrals remain a significant obstacle.^1^

A key factor in COPD healthcare inequities is the distribution of NHS resources.^2^ Hospitalization rates are often lower in areas with greater healthcare financing and more comprehensive specialized services.^3^ Despite high illness incidence, certain regions suffer from persistent underfunding, which increases their dependency on hospital and emergency services.^4^

## The impact of policy, primary care access, and geography on COPD outcomes

Clinical judgment at the CCG level significantly influences care variation. Restrictive regulations on prescription patterns and expert referrals in some areas make it more difficult to manage COPD effectively, which increases the strain on hospitals.^5^ There are gaps in post discharge treatment because pulmonary rehabilitation programs, which have been demonstrated to enhance patient outcomes dramatically, are not consistently provided throughout England.^6^

**Fig. 1:**
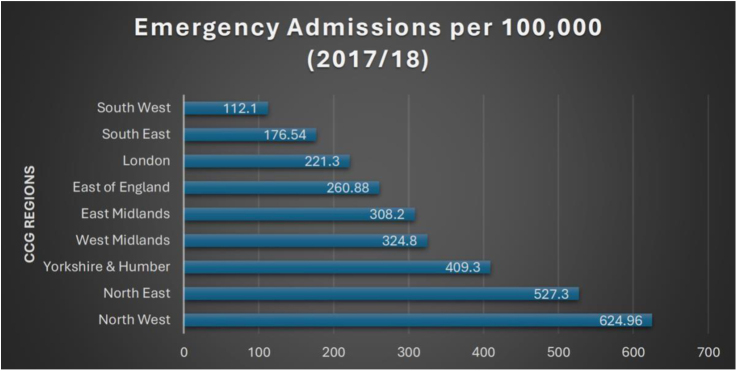
COPD 30-day readmission rates across CCGs (2017/18). Variation in COPD emergency hospital admission rates across Clinical Commissioning Groups (CCGs) in England, 2017/18.

*Source*: Public Health England. The 2nd Atlas of Variation in Risk Factors and Healthcare for Respiratory Disease in England (2020); author calculations.^1^ COPD Emergency Admission Rates Across CCGs (2017/18).

The geographical differences in COPD emergency hospital admission rates per 100,000 population among CCGs in England are graphically depicted in Fig. 1. The graph illustrates how admittance rates are noticeably higher in northern regions, especially in the North West and North East as compared to the southern regions.^1^, ^6^

This variance raises the possibility of restrictions in primary care accessibility, preventive care provision, and early intervention tactics. The management of COPD may be more hospital-centered than community-based in areas with greater emergency admission rates, which might result in higher healthcare expenditures and worse long-term results.^8^ On the other hand, areas like the South West that have lower admission rates could have better outpatient illness management, more robust primary care systems, and more successful preventative care programs.

Accessibility to primary care is another important factor. Patients in underprivileged regions are more likely to experience exacerbations that necessitate emergency treatments because they frequently face longer wait times and fewer preventative care options.^7^ Prompt access to respiratory experts improves illness management and lowers unnecessary hospital admissions by ensuring accurate diagnoses and the best possible treatment options.^8^ Geographic considerations also influence healthcare delivery. Additional obstacles, such as poor local healthcare facilities and transportation, worsen COPD care disparities in rural and socioeconomically disadvantaged areas.^9^ To address these problems, strategic resource allocation and policy changes targeted at enhancing healthcare access in all areas are needed.

## Conclusion and discussion

Reducing regional healthcare inequalities in England requires increasing access to prompt, high-quality COPD treatment services. This issue brief's findings demonstrate how disparities in patient outcomes are influenced by policy decisions, primary care access, and specialist availability.^6^ Preventive strategies, better care coordination, and equitable resource allocation must all be included into a multidimensional strategy to address these issues.^7^ Health authorities may improve COPD outcomes and lower avoidable hospitalizations by emphasizing early diagnosis, growing pulmonary rehabilitation programs, and bolstering community-based treatment.^3^ Furthermore, a higher degree of integration between hospital and community care settings can lower the risk of readmission by ensuring that patients who are released receive proper follow-up treatment.^4^ The gap between inpatient and outpatient settings should be further closed by bolstering transitional care initiatives, such as specialized COPD case managers.^5^

Furthermore, reducing supply-sensitive care differences and enhancing overall healthcare equality would need focused actions in high-risk locations.^8^ Prioritizing healthcare budget and labor allocation should go to areas with persistently high hospitalization rates for COPD.^9^ Reducing COPD-related morbidity will also require addressing socioeconomic determinants of health, such as smoking prevalence, air pollution, and economic stability.^2^ Incorporating COPD-specific quality indicators into compensation structures may incentivize healthcare professionals to prioritize long-term disease management and early therapies as healthcare systems transition to value-based care models.^1^

## Policy implications

Policymakers in the NHS should prioritize reallocating resources to high-admission areas to reduce supply-sensitive care variation, ensuring that spending is in line with the burden of disease.^4^ Hospitalization rates can be decreased, and early intervention can be made easier by improving primary care involvement through GP training programs and better diagnostic equipment.^5^ Expanding pulmonary rehabilitation programs will result in lower readmission rates and improved post-discharge.^6^ Finally, a more equal healthcare system may be ensured using real-time hospital admission data to inform policy changes and resource allocation.^7^ A diversified strategy is needed to address geographical differences in COPD therapy. NHS England can improve COPD outcomes and lessen the burden on emergency services by enacting specific policy changes and expanding access to healthcare.^8^

## Authors’ contributions

SS: conceptualisation, investigation, writing original draft, writing and editing, final approval.

## Informed consent

This study used publicly available secondary aggregate data and did not involve human participants or identifiable personal information. Therefore, ethical approval and informed consent were not required.

## Funding

This research received no specific grant from any funding agency in the public, commercial, or not-for-profit sectors.

## Generative AI use

Not used for the manuscript creation.

## Conflicts of interest

The author declares no conflict of interests.

## References

[1] Public Health England (2020).

[2] Kim M., Ren J., Tillis W., Asche C., Kim I., Kirkness C. (2016). Explaining the link between access-to-care factors and health care resource utilization among individuals with COPD. Int J Chronic Obstruct Pulmon Dis.

[3] Sohanpal R., Pinnock H., Steed L., Heslop-Marshall K., Kelly M.J., Chan C. (2024). A tailored psychological intervention for anxiety and depression management in people with chronic obstructive pulmonary disease: tandem RCT and process evaluation. Health Technol Assess.

[4] Sampson F., O’cathain A., Strong M., Pickin M., Esmonde L. (2012). Commissioning processes in primary care trusts: a repeated cross-sectional survey of Health Care Commissioners in England. J Health Serv Res Policy.

[5] Boulieri A., Hansell A., Blangiardo M. (2016). Investigating trends in asthma and COPD through multiple data sources: a small area study. Spat Spatio-temp Epidemiol.

[6] Sinha I.P., Calvert J., Hickman K.C., Hurst J.R., McMillan V., Quint J.K. (2019). National asthma and COPD audit programme and the NHS Long Term Plan. Lancet Respir Med.

[7] Burch P., Doran T., Kontopantelis E. (2018). Regional variation and predictors of overregistration in English primary care in 2014: a spatial analysis. J Epidemiol Community Health.

[8] Takundwa R., Jowett S., McLeod H., Peñaloza-Ramos M.C. (2017). The effects of environmental factors on the efficiency of Clinical Commissioning Groups in England: A Data Envelopment analysis. J Med Syst.

[9] Tian J., McGrogan A., Jones M.D. (2022). Temporal and geographical variation in low carbon inhaler dispensing in England, 2016 to 2021: An ecological study. J R Soc Med.

